# Opioids in patients with COPD and refractory dyspnea: literature review and design of a multicenter double blind study of low dosed morphine and fentanyl (MoreFoRCOPD)

**DOI:** 10.1186/s12890-021-01647-8

**Published:** 2021-09-10

**Authors:** Marlies van Dijk, Kris J. M. Mooren, Jan-Willem K. van den Berg, Wendy J. C. van Beurden-Moeskops, Roxane Heller-Baan, Sander M. de Hosson, Wai Yee Lam-Wong, Liesbeth Peters, Karin Pool, Huib A. M. Kerstjens

**Affiliations:** 1 Department of Pulmonary Diseases, University of Groningen, University Medical Center Groningen, Home Postal Code AA11, PO Box 30001, 9700 RB Groningen, The Netherlands; 2 Groningen Research Institute of Asthma and COPD (GRIAC), University of Groningen, Hanzeplein 1, 9713 RB Groningen, The Netherlands; 3 Department of Pulmonary Diseases, Spaarne Ziekenhuis, Boerhaavelaan 22, 2035 RC Haarlem, The Netherlands; 4 Department of Pulmonary Diseases, Isala Klinieken, Dokter van Heesweg 2, 8025 AB Zwolle, The Netherlands; 5 Department of Pulmonary Diseases, Medisch Spectrum Twente, Koningsplein 1, 7512 KZ Enschede, The Netherlands; 6 Department of Pulmonary Diseases, Ikazia Ziekenhuis, Montesorriweg 1, 3083 AN Rotterdam, The Netherlands; 7 Department of Pulmonary Diseases, Wilhelmina Ziekenhuis Assen, Europaweg-Zuid 1, 9400 RA Assen, The Netherlands; 8 Department of Pulmonary Diseases, Elkerliek ziekenhuis, Wesselmanlaan 25, 5707 HA Helmond, The Netherlands; 9 Department of Pulmonary Diseases, Noordwest Ziekenhuisgroep, Wendelaarstraat 58, 1814 GS Alkmaar, The Netherlands; 10 Department of Pulmonary Diseases, Rode Kruis Ziekenhuis, Vondellaan 13, 1942 LE Beverwijk, The Netherlands

**Keywords:** COPD, Refractory dyspnea, Breathlessness, Opioids

## Abstract

**Background:**

Refractory dyspnea or breathlessness is a common symptom in patients with advanced chronic obstructive pulmonary disease (COPD), with a high negative impact on quality of life (QoL). Low dosed opioids have been investigated for refractory dyspnea in COPD and other life-limiting conditions, and some positive effects were demonstrated. However, upon first assessment of the literature, the quality of evidence in COPD seemed low or inconclusive, and focused mainly on morphine which may have more side effects than other opioids such as fentanyl. For the current publication we performed a systematic literature search. We searched for placebo-controlled randomized clinical trials investigating opioids for refractory dyspnea caused by COPD. We included trials reporting on dyspnea, health status and/or QoL. Three of fifteen trials demonstrated a significant positive effect of opioids on dyspnea. Only one of four trials reporting on QoL or health status, demonstrated a significant positive effect. Two-thirds of included trials investigated morphine. We found no placebo-controlled RCT on transdermal fentanyl. Subsequently, we hypothesized that both fentanyl and morphine provide a greater reduction of dyspnea than placebo, and that fentanyl has less side effects than morphine.

**Methods:**

We describe the design of a robust, multi-center, double blind, double-dummy, cross-over, randomized, placebo-controlled clinical trial with three study arms investigating transdermal fentanyl 12 mcg/h and morphine sustained-release 10 mg b.i.d. The primary endpoint is change in daily mean dyspnea sensation measured on a numeric rating scale. Secondary endpoints are change in daily worst dyspnea, QoL, anxiety, sleep quality, hypercapnia, side effects, patient preference, and continued opioid use. Sixty patients with severe stable COPD and refractory dyspnea (FEV_1_ < 50%, mMRC ≥ 3, on optimal standard therapy) will be included.

**Discussion:**

Evidence for opioids for refractory dyspnea in COPD is not as robust as usually appreciated. We designed a study comparing both the more commonly used opioid morphine, and transdermal fentanyl to placebo. The cross-over design will help to get a better impression of patient preferences. We believe our study design to investigate both sustained-release morphine and transdermal fentanyl for refractory dyspnea will provide valuable information for better treatment of refractory dyspnea in COPD.

*Trial registration* NCT03834363 (ClinicalTrials.gov), registred at 7 Feb 2019, https://clinicaltrials.gov/ct2/show/NCT03834363.

**Supplementary Information:**

The online version contains supplementary material available at 10.1186/s12890-021-01647-8.

## Background

Refractory dyspnea or breathlessness is a common symptom in patients with advanced chronic obstructive pulmonary disease (COPD), with a prevalence of up to 94% in the last year of life [1, 2]. It is defined as persisting complaints of dyspnea despite optimal standard therapy including, but not limited to smoking cessation, education, inhaled bronchodilators and pulmonary physiotherapy [3]. Refractory dyspnea is known to severely impact quality of life and exercise tolerance, and to increase the risk of depression and anxiety [4]. As the prevalence of COPD is expected to rise during the upcoming decades [5], it is likely that the number of patients with COPD suffering from refractory dyspnea will also continue to grow.

Advanced treatments such as non-invasive ventilation, bronchoscopic lung volume reduction and lung transplantation can improve dyspnea and quality of life in patients with advanced COPD [6]. But these treatments are only available for a proportion of patients with advanced COPD, due to strict eligibility criteria, high health-care costs and sometimes scarcity. Therefore, there is still a need for more widely available treatments of refractory dyspnea. In this context low dosed opioids have previously been investigated, and some positive effect was demonstrated [7–9]. However, whether the quality of the evidence is sufficient is still a topic of discussion. Furthermore, despite a positive advice on opioids in palliative care guidelines for COPD, prescription appears to be low in clinical practice [10–12].

We performed a systematic literature search with respect to opioids for refractory dyspnea in COPD, which we updated for the current publication to include all recent trials. We searched for placebo-controlled randomized clinical trials investigating any type of opioid prescribed for dyspnea reduction in COPD (at least 50% of participants). We included trials reporting on dyspnea, health status and/or quality of life. Additional details on the search strategy can be found in the online supplement (Additional file 1: Online supplement MoreFoRCOPD), including a flow chart on the number of records identified, screened and included.

Table 1 shows an overview of the trials we identified as a result of our search strategy. In total, fifteen trials were included. A statistically significant positive effect on dyspnea of opioid versus placebo was demonstrated only in three studies [7, 8, 13]. Since the majority of these studies included a small number of patients, the lack of statistically significant results may in part be explained by a low statistical power to detect a treatment effect. This assumption is supported by a meta-analysis published by Ekström et al. in 2015, in which a positive effect on dyspnea was found for both systemically administered and nebulized opioids (analyses of combined data of 8 and 4 trials, respectively) [14]. Nevertheless, the three largest studies in our table, which all have been published more recently, demonstrated no significant change in dyspnea for sustained-release morphine and oxycodone [15–17]. While assessing this, it is important to note that in the studies of Currow et al. [15] and Ferriera et al. [16] (which were originally both part of a three-armed trial) all arms received immediate-release morphine as needed. For both studies, the immediate-release morphine was used significantly more frequently in the placebo group (8.7 vs. 5.8 and 7.0 vs. 4.2 daily doses, respectively) [15, 16] making an overall effect of the maintenance morphine more difficult to detect. Furthermore, in the study by Verberkt et al. there was a statistically significant effect on worst daily dyspnea measured on a numeric rating scale (NRS) in a subgroup of COPD patients with a modified Medical Research Council (mMRC) ≥ 3 (mean difference compared to placebo: − 1.33 (− 2.50 to − 0.16) points) [17].

**Table 1:** Overview of randomized clinical trials investigating the effect of opioids on dyspnea, quality of life or health status in COPD

References	Design	n (% COPD)	Setting	Comparison	Treatment duration	Breathlessness			Quality of life or health status		
						Measurement (scale)	Opioid	Placebo	Measurement (scale)	Opioid	Placebo
Woodcock et al. [18]	Cross-over	12 (100)	Outpatient	Dihydrocodeine	Single dose	VAS (0–10 cm) 45 min after med	5.54 ± 1.91	6.33 ± 2.0	–	–	–
Light et al. [19]	Cross-over	13 (100)	Outpatient	Oral morphine 0.8 mg/kg	Single dose	Borg score (0–10) Rest	0.29 ± 0.58	0.13 ± 0.23	–	–	–
Jankelson et al. [20]	Cross-over	16 (100)	Outpatient	Nebulized morphine 20/40 mg	Single dose	Borg score (0–10) After 6MWT	4.2 ± 2.1/4.3 ± 1.8	4.3 ± 2.2	–	–	–
Noseda et al. [21]	Cross-over	14 (79)^#^	Hospitalized	Nebulized morphine 10/20 mg ± oxygen	Single dose	VAS (− 100 to + 100%)	+ 33 ± 28/+ 43 ± 27	+ 42 ± 27	–	–	–
Jensen et al. [22]	Cross-over	12 (100)	Outpatient	Nebulized fentanyl 50 µg	Single dose	Borg score (0–10) Isotime CPET	2.0 ± 0.5	2.6 ± 0.5	–	–	–
Abdallah et al. [13]	Cross-over	20 (100)	Outpatient	Morphine dose up to 10 mg	Single dose	Borg score (0–10) Isotime CPET	3.0 ± 1.6*	4.2 ± 2.6	–	–	–
Iupati et al. [23]	Cross-over Multicenter	21 (62)	Outpatient	Intranasal fentanyl 20 µg as needed	1 day	VAS (0–100 mm) 15 min after med	26 ± 21 (Δ29 ± 25)	21 ± 19 (Δ33 ± 24)	–	–	–
Abernethy et al. [7]	Cross-over Multicenter	48 (88)^#^	Outpatient	SR morphine 20 mg od	4 days	VAS (0–100 mm) Morning/evening	40.1 ± 24*/40.3 ± 23*	47.7 ± 26 49.9 ± 24	Data not presented	Data not presented	Data not presented
Janowiak et al. [24]	Cross-over	10 (100)	Hospitalized	Nebulized morphine 3–5 mg qid	4 days	VAS (0–100 mm) Now (2dd)	Δ25.4 ± 9.0^$^	Δ6.3 ± 7.8	–	–	–
Johnson et al. [8]	Cross-over	18 (100)	Outpatient	Dihydrocodeine 15 mg as needed up to tds	7 days	VAS (0–10 cm) Mean daily	4.6 ± 2.1*	5.6 ± 2.3	–	–	–
Currow et al. [15]	Parallel Multicenter	284 (58)^#^	Outpatient	SR morphine 20 mg qd All arms: morphine 2.5 mg as needed	7 days	VAS (0–100 mm) Now (2dd)	Δ-5.00 ± 2.13	Δ-4.86 ± 2.07	EORTC-QLQ-C15 PAL (0–100)	Δ1.8 ± 2.2	Δ1.5 ± 2.2
Ferriera et al. [16]	Parallel Multicenter	155 (60)^#^	Outpatient	Oxycodone 5 mg tds All arms: morphine 2.5 mg as needed	7 days	VAS (0–100 mm) Now	Δ-3.7 ± 2.9	Δ-9.0 ± 2.7	EORTC-QLQ-C15 PAL (0–100)	Δ-1.7 ± 3.1	Δ2.82 ± 3.1
Eiser et al. [25]	Cross-over	14 (100)	Outpatient	Diamorphine 2.5/5 mg qid	14 days	VAS (0–10 cm)	7.0 ± 0.7/7.0 ± 0.8	6.5 ± 0.7	–	–	–
Verberkt et al. [17]	Parallel Multicenter	124 (100)	Outpatient	SR morphine 10 mg 1-tds	28 days	NRS (0–10 points) Mean	Δ-0.60 (− 1.55 to 0.35)		CAT (0–40)	Δ-2.18 (− 4.14 to − 0.2)*	
Poole et al. [9]	Cross-over	16 (100)	Outpatient	SR morphine 10 mg od or bid	42 days	DBS (0–5)	2.22	2.26	CRQ (20–140)	∆2.08 ± 4.53	∆2.94 ± 3.46

Data presented as mean ± SD

*od* once a day, *bid* twice daily, *tds* three times a day, *qid* four times a day, *SR* sustained release, *VAS* visual analogue score, *DBS* daytime breathlessness score, *NRS* numeric rating scale, *CRQ* chronic respiratory questionnaire, *EORTC-QLQ-C15 PAL* Quality of life questionnaire developed by the European Organisation for Research and Treatment of Cancer, *CAT* COPD assessment test, *CPET* cardiopulmonary exercise testing, *6MWT* 6-min walking test

*p < 0.05 opioid versus placebo, ^$^*p* < 0.05 change after treatment. ^#^Data not exclusively on COPD

Information on quality of life or health status was limited to four RCT’s. Of these, only the study by Verberkt et al. demonstrated a small positive, statistically significant effect on health status measured with the COPD assessment test (CAT) [17]. Our search identified no placebo-controlled RCT’s investigating transdermal fentanyl for refractory dyspnea in COPD.

Based on this assessment of available evidence, we designed a randomized, placebo-controlled clinical trial, on which we will further elaborate in the “Methods/design” section and “Discussion” section.

## Methods/design

### Overview

We designed a robust, multi-center, double blind, double-dummy, cross-over, randomized, placebo-controlled clinical trial with three study arms investigating transdermal fentanyl and sustained-release morphine. We hypothesize that both fentanyl and morphine provide a reduction of dyspnea which is greater than placebo, and that fentanyl has less side effects than morphine. A total of 60 patients with severe stable COPD and refractory dyspnea will be included in this study in ten Dutch hospitals. Patients will be recruited at the outpatient clinic of each participating hospital by chest physicians. The study is registered at clinicaltrials.gov (NCT03834363), where a full list of participating hospitals can be found, and the protocol is approved by the UMCG Ethics committee. Written informed consent will be obtained from all participants and the study will be performed in accordance with the Declaration of Helsinki.

### Study duration and treatment

**Fig. 1:**
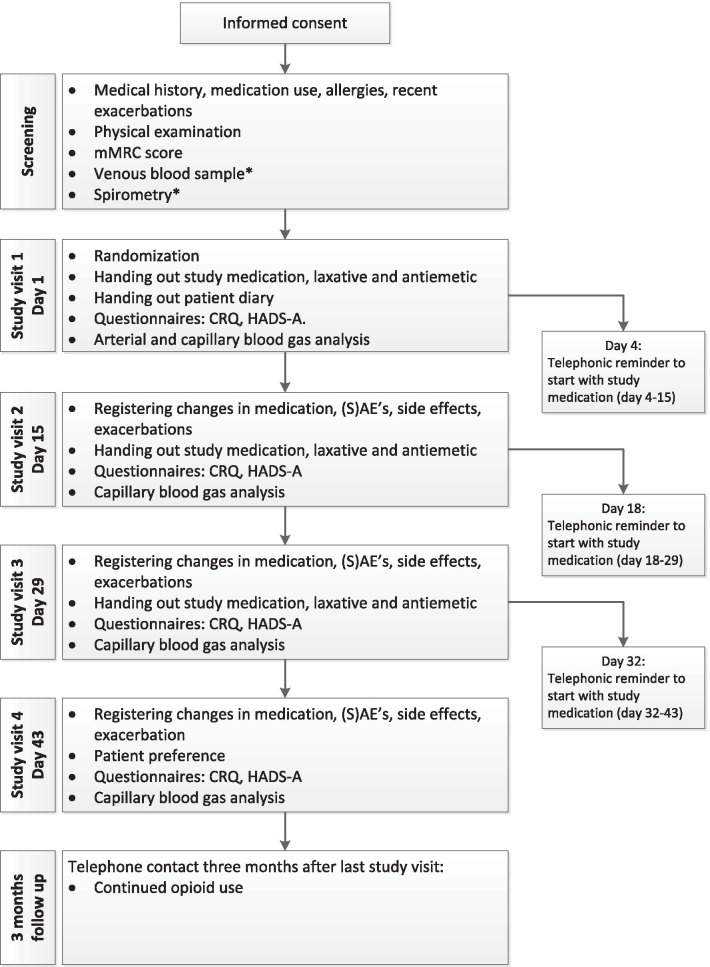
Study flowchart. *mMRC* modified Medical Research Council Score, *CRQ* chronic respiratory questionnaire, *HADS-A* hospital anxiety depression score—anxiety, *(S)AE* (serious) adverse event. *Unless already performed in the 6 months before screening

The study duration is 6 weeks for each participant, divided in three periods of 2 weeks. During each period the participant is treated for 11 days. During the first 3 days of every treatment period no study medication is used, to wash out medication of any previous treatment period. The fentanyl patches are dosed 12 µg/h and changed every 3 days. The morphine sustained-released capsules are dosed 10 mg b.i.d. Both an antiemetic (metoclopramide 10 mg as needed, up to thrice daily) and laxative (macrogol/electrolytes 13.7 g, started once daily, more or less sachets as needed) are prescribed. In total, there are four study visits. A complete study flowchart can be found in Fig. 1. After the end of the study treatment patients can discuss with their chest physician whether they would like to continue with low dosed morphine or transdermal fentanyl. At the time of this decision, the participants and physician are still blinded to the study treatment.

### In- and exclusion criteria

All in- and exclusion criteria can be found in Table 2. In general, patients with COPD Gold class III or IV and a modified Medical Research Council score (mMRC) ≥ 3 who perceive dyspnea despite optimal standard therapy according to GOLD and the Dutch guideline for diagnosis and treatment of COPD can be included. If there is comorbidity substantially contributing to the breathlessness, for example severe heart failure, patients are excluded. Participants who have a moderate or severe exacerbation (requiring oral corticosteroids, antibiotics and/or hospital admission) during participation are discontinued from the trial. If they are stable for 8 weeks after recovery from the exacerbation, they are allowed to restart the study once more.

**Table 2:** In- and exclusion criteria

*Inclusion criteria*
Age ≥ 40 years
Read, understood and signed the Informed Consent form
COPD GOLD class III or IV, according to GOLD criteria
Post-bronchodilatation FEV1/FVC ≤ 70% and FEV1 < 50% pred.*
Complaints of refractory dyspnea as established by patient and doctor
mMRC score ≥ 3
Life expectancy of ≥ 2 months
Optimized standard therapy according to Dutch LAN guideline for diagnosis and treatment of COPD
*Exclusion criteria*
Other severe disease with chronic pain or chronic dyspnea (a non-susbstantial component of left sided heart failure is acceptable)
Current use of opioids for whatever indication
Allergy/intolerance for opioids
Psychiatric disease, not related to severe COPD
Exacerbation of COPD 8 weeks prior to inclusion or between screening and randomization
Problematic (leading to medical help or social problems) substance abuse during the last 5 years
Active malignancy, with the exception of planocellular or basal cell carcinoma of the skin
eGFR < 15 ml/min*

*Measured within 6 months of screening

*COPD* chronic obstructive pulmonary disease, *FEV1* forced expiratory volume in 1 s, *FVC* forced vital capacity, *GOLD* global initiative for chronic obstructive lung disease, *LAN* Lung Alliance The Netherlands, *mMRC* modified Medical Research Council Dyspnea Scale, *eGFR* estimated Glomerular Filtration Rate

### Outcome measurements

The primary outcome measurement is change in mean daily dyspnea sensation as measured on the numeric rating scale for Dyspnea [26]. Secondary outcome measurements are change in worst daily dyspnea sensation, health-related quality of life, anxiety, sleep quality, occurrence of respiratory depression and side effects, patient preference and continued opioid use. A more extensive description of the outcome measures can be found in Table 3. Patients who drop out will be followed as much as possible for vital status, hospitalization, and start of open label opioids during the intended 6 weeks period of the study.

**Table 3:** Outcome measurements

	Measurement	Frequency of measurement
*Primary outcome measure*		
Change in mean dyspnea sensation	Numeric rating scale [26]	Once daily in patient diary
*Secondary outcome measures*		
Change in worst dyspnea sensation	Numeric rating scale [26]	Once daily in patient diary
Change in Health-Related Quality of Life	CCQ [27]	Once daily in patient diary
	CRQ [28]	During each study visit
	CRQ-mastery domain	During each study visit
	HADS-A [29] Open en named side effects	During each study visit Once daily in patient diary and asked during study visits
Anxiety Side effects Change in hypercapnia, HCO_3_ and pH Change in sleep quality Patient preference Continued opioid use	Capillary blood gas analysis Numeric rating scale [30] Asked during the final study visit Asked 3 months after the end of study	During each study visit Once daily in patient diary Once Once

*CCQ* clinical COPD questionnaire, *CRQ* chronic respiratory questionnaire, *HADS-A* hospital anxiety and depression scale—anxiety subscale

### Randomization and unblinding

Randomization is tailor made for this study using a web based program (ALEA^®^ DM version 17.1). Randomization can be performed online by the research team of each participating hospital. Participants will be randomized equally between the six possible treatment sequences, stratified for study location. Unblinding only occurs in the case of patient emergencies and at the conclusion of the study. Health authorities will be granted access to unblinded data if needed. The pharmacist on call of each participating hospital can unblind a participant using the web based program if requested by the researcher because of a patient emergency.

### Statistical analysis

For the power calculation the difference in primary endpoint between fentanyl and placebo was used. The Minimal Clinical Important Difference (MCID) for the NRS score is 1 point, the standard deviation is 2.0 points [31]. With a two-sided alpha = 0.05 and a power of 0.90 in a cross-over design, 44 participants who complete the study are needed. Because this is a fragile patient group, we will aim to recruit 60 participants.

The primary endpoint analysis will be on an intention to treat basis and therefore all patients randomized. The primary endpoint is the NRS mean dyspnea score which we will treat as a continuous variable for day 7–14. This will not be calculated if less dan 2 days are available. Since it is a three way cross-over, the data for the available periods will also be used of not all periods were completed. No imputation will be used for the primary endpoint. There will be two comparisons: the difference in the mean dyspnea score of day 7–14 for fentanyl versus placebo and for morphine versus placebo. In this way, the risk of any remaining effect from the previous treatment periods influencing the outcome will be optimally reduced. The analysis will be by Student’s t-test. The analyses of secondary endpoints will be done by Student’s t-tests (or non-parametric tests where needed) or chi square, following the same scheme of main comparisons as for the primary endpoints. The analysis of side effects will be done by comparison of proportions of side effects by chi square tests between all three arms. Composite questionnaire data will be primarily analysed by total sum scores. Additionally, per protocol analyses will be performed. The study is not powered to determine equivalence of dyspnea relief of fentanyl compared to morphine: that comparison will consist of descriptive statistics only.

### Safety

All (serious) adverse events will be monitored. The sponsor will report serious adverse events (SAEs) through the Dutch web portal *ToetsingOnline* to the accredited Ethics committee that approved the protocol, within 7 days of first knowledge for SAEs that result in death or are life threatening followed by a period of maximum of 8 days to complete the initial preliminary report. All other SAEs will be reported within a period of maximum 15 days after the sponsor has first knowledge of the serious adverse events. This is a short term study with 60 patients, entered parallel in a multi-centre study. Therefore, and since opioids in the form of morphine are in the guidelines, we will not perform interim analyses, even though the patient population of patients with severe COPD and in a palliative setting is at increased risk of death. For the same reasons, no Data Safety Monitoring Board (DSMB) will be instituted.

### Study timeline

The study has started in November 2019. At this point the first participant was included at the University Medical Center Groningen. For the other participating hospitals the start of inclusion was delayed by one or more months because of a delay in the production of research medication and a delay in the issuing of a permit for scientific research with opioids for the participating hospital pharmacies. Unfortunately, starting March 2020 the inclusion was alternately put on hold or restricted in each participating hospital due to the COVID-19 pandemic. We aim to include all patients by the end of 2021, but whether this will be achieved is strongly depended on the course of the COVID-19 pandemic.

## Discussion

Optimal reduction of dyspnea in patients with severe COPD is an important way to improve quality of life, yet can be very challenging. From our assessment of the literature, we found that even though opioids have found their way into COPD guidelines as a treatment option for refractory dyspnea, the evidence base can still be considered inconclusive. Furthermore, the majority of research has focused on morphine and we identified no placebo-controlled RCT investigating transdermal fentanyl. However, trials investigating fentanyl in the short-acting form, suggest that fentanyl could give a reduction of dyspnea [32, 33]. Additionally, studies on pain treatment indicate that patients may prefer transdermal fentanyl and experience less side effects in comparison to oral morphine [34]. Therefore, we believe that our current multi-center, double blind, cross-over, placebo-controlled study design to investigate sustained-release morphine and transdermal fentanyl for refractory dyspnea will provide valuable information on patient preference and the effectiveness of transdermal fentanyl and sustained-release morphine for refractory dyspnea in COPD.

By choosing a cross-over design for this study the participant is his or her own control, thus reducing the variability and the number of patients needed to participate. Additionally, this design helps to get a better impression of patient preferences. On the other hand, because of the cross-over design the treatment duration is 6 weeks instead of 11 days (which it would be if this study had a parallel design). This prolonged study duration will most likely increase the risk of participants that have to be discontinued from the trial because of the occurrence of COPD exacerbations, which occur frequently in advanced COPD. For this reason we aim to include 60 participants, which is sixteen more than the 44 participants calculated from the power analysis which need to fully complete the study. Furthermore, patients experiencing an exacerbation will discontinue the trial, but may be included once more if they are clinically stable for at least 8 weeks.

There are indications that prescription of opioids for refractory dyspnea in COPD can be a loaded topic for both patient and doctors, amongst others because of associations with terminal disease, possible adverse effects and addiction [10]. Although this has not been formally investigated in patients, we believe education is important to address any questions or worries patients may have regarding opioids. Therefore, both an animated short film for patients and their loved ones on facts and myths about opioids (developed by Indiveo B.V.) as well as an information leaflet with the same content are tested during our study. At the end of the trial, feedback from the participants will be used to adjust the animation and leaflet and these will be made widely available for patients with COPD. Additionally, both patients and physicians participating in the study are asked to share their experiences with opioids for refractory dyspnea in COPD during regional congresses and meetings.

## Supplementary Information

**Additional file 1.** Online supplement MoreFoRCOPD.

## Data Availability

The data management plan is made available in de the online supplement (Additional file 1: Online supplement MoreFoRCOPD). Marlies (M.) van Dijk or Huib (H.A.M.) Kerstjens can be contacted to apply for permission to obtain access to the raw data that will be generated during the study.

## Abbreviations

AE: Adverse event
CCMO: Central Committee on Research Involving Human Subjects
CCQ: Clinical COPD Questionnaire
CRQ: Chronic Respiratory Questionnaire
COPD: Chronic obstructive pulmonary disease
eGFR: Estimated Glomerular Filtration Rate
FEV1: Forced expiratory volume in 1 s
FVC: Forced vital capacity
GOLD: Global initiative for chronic obstructive lung disease
HR-QoL: Health Related Quality of Life
HADS-A: Hospital Anxiety and Depression Scale—anxiety subscale
IC: Informed consent
LAN: Lung Alliance The Netherlands
mMRC: Modified Medical Research Council Dyspnea Scale
MREC: Medical Research Ethics Committee
NRS: Numeric rating scale
SAE: Serious adverse event
SR: Sustained-release
