# Supplementary material for: Opioids in patients with COPD and refractory dyspnea: literature review and design of a multicenter double blind study of low dosed morphine and fentanyl (MoreFoRCOPD)
Source: BMC Pulm Med. 2021 Sep 10;21:289. doi: 10.1186/s12890-021-01647-8 (PMC8431258; doi:10.1186/s12890-021-01647-8)
Supplement: Supplementary file 1 — Additional file 1. Online supplement MoreFoRCOPD. [file 12890_2021_1647_MOESM1_ESM.docx]

**Online supplement to ‘Design for a multicenter, double blind, double-dummy cross-over randomized placebo controlled clinical trial to evaluate the effect of low dosed morphine and fentanyl on refractory dyspnea in COPD (MoreFoRCOPD)’**

**Content**

**Page number**

1. Search strategy 2
2. Figure S1: Flow chart of search strategy 3
3. Data management plan 4
4. **Search strategy**

Study design: Placebo-controlled randomized clinical trials.

Study treatment: Any type of opioid prescribed for dyspnea reduction.

Study population: COPD patients (≥ 50% of total number of participants)

Study endpoints: Dyspnea, quality of life and/or health status.

PubMed search:

Performed on: 26^th^ March, 2021.

("morphine"[Title/Abstract] OR "codeine"[Title/Abstract] OR "fentanyl"[Title/Abstract] OR "dihydrocodeine"[Title/Abstract] OR "diamorphine"[Title/Abstract] OR "Oxycodone"[Title/Abstract] OR "Hydrocodone"[Title/Abstract] OR "Methadone"[Title/Abstract] OR "buprenorphine"[Title/Abstract] OR "meperidine"[Title/Abstract] OR "hydromorphone"[Title/Abstract] OR "oxymorphone"[Title/Abstract] OR "tramadol"[Title/Abstract] OR "carfentanil"[Title/Abstract]) AND ("COPD"[Title/Abstract] OR "Chronic obstructive"[Title/Abstract] OR "airflow obstruction"[Title/Abstract] OR "dyspn*"[Title/Abstract] OR "breathlessness"[Title/Abstract])

1. Flowchart

1. **Data management plan**

| **SUMMARY** | |
| --- | --- |
| **Project name, title, date and version:** | Morphine or Fentanyl for Refractory Dyspnea in COPD (MoreFoRCOPD), 11-10-2019, version 1.0 |
| **Subsiding party(ies) + grant number** | Innovatiefonds Zorgverzekeraars  Stichting Astmabestrijding |
| **Coordinating investigator/project leader** | Prof. dr. H.A.M. Kerstjens |
| **Partner organisations (if applicable):** | OZG; WZA; Isala Klinieken; MST; Elkerliek zkh; Ikazia zkh; NWZ; RKZ Beverwijk; Spaarna Gasthuis. |
| **Project duration:** | Start: **01-12-2019**  End: **01-12-2020 (adjusted to 31-12-2021)** |
| **Objectives:** | We will investigate the following hypothesis: Both Fentanyl and morphine provide a reduction of dyspnea which is better than placebo. Fentanyl has less side effects than morphine. |
| **Primary endpoints:** | The primary endpoint is change in dyspnea sensation. |
| **Secondary endpoints (if applicable):** | Secondary endpoints are change in HR-QoL, anxiety, sleep quality, hypercapnia and the number and seriousness of side effect. |

| **ROLES AND RESPONSIBILITIES DATAMANAGEMENT**  *Please provide names of institutions/parties participating in data management for this research* | |
| --- | --- |
| **Data collection:** | Research nurse and/or principal investigator of every site. |
| **Data quality control:** | Research nurse UMCG (data manager) |
| **Data processing:** | Principal investigator UMC Groningen (PhD project) |
| **Data analysis:** | Principal investigator UMC Groningen (PhD project) |
| **Data archiving:** | Research nurse and/or principal investigator of every site for local data.  Principle investigator UMCG for central, anonymised data storage . |

# Data description

Each subject will receive a study ID. The code will not be based on the patient initials and birth date, but on number of enrolment into the study. The subject identification code list will be controlled by the Principal Investigator.

All data is newly collected, since these patients will be newly included in this study.

We will collect data on baseline characteristics such as age (not date of birth), gender, smoking status and medication use.

We will collect patient diaries which will contain questionnaires (NRS dyspnea, sleep, CCQ) and information about side effects of medication.

We will collect two questionnaires (CRQ, HADS-A) during the study visits.

We will collect the data (results) of a spirometry, venous blood sampling, arterial blood gas analysis and capillary blood gas analysis. Blood samples (venous, arterial, capillary) will not be stored for the study.

Al questions of the questionnaires will be filled out separately in the electronic Case Report Forms. In the final (locked) database, the raw data will be processed to questionnaire scores (total score, and subdomains if applicable).

The research nurse or principal investigator of each site will fill out all data in an electronic CRF.

A data manager will control quality of data. There will be a monitor for control of data processing.

# Data storage and archiving

All primary data will be keyed in RedCap in a web-based version with password protected entry. These data are stored on an online server hosted by the UMCG. Anonymized data in the analysis phase will be stored digitally at the University Medical Center Groningen (UMCG) network

< G:\Specifieke sites, onderafdelingen en research\Astma COPD research\MoreFoRCOPD> .

This data storage environment allows for careful access management and is only accessible with username and password and is compliant with UMCG policies. Every workstation of the UMCG is part of a standard IT-workplace which is backed-up automatically every day on the servers of the UMCG. The non-digital data (e.g. paper questionnaires) will be stored in a locked archive at the study site where the data was collected. Management of the data and access permissions are with the data manager of the research project. The raw data containing identifiable information will be kept strictly separate from the processed data.

The digital data will not exceed 15 GB and will be stored for at least 15 years after publication of the main results. The basic research and storage infrastructure is accounted for by the UMCG.

Data documentation and metadata

A description of the data collection will be documented in the UMCG Research Register. A UMCG Research Register Identifier will be assigned. The data will be reported in eCRFs in the online program RedCap (provided by the UMCG), where a back up is available.

# Data access and re-use

Access permissions are with the project leader of the research project. The raw data containing identifiable information will be kept strictly separate from the processed data and can only be accessed by the data manager and the PI. After publication of the main results, the processed, pseudonymised data will be made available for re-use. Requests for re-use of data will be evaluated by the project leader who will check whether the research question falls within the scope of the informed consent. Third party use of data is governed in part by IP rules and agreements, that will become available after publication of the main results.
